# Non-GDANets: Sports small object detection of thermal images with Non-Glodal decoupled Attention

**DOI:** 10.1371/journal.pone.0270376

**Published:** 2022-07-06

**Authors:** Jia Zhao, Bingfei Mao, Hengran Meng, Liping Wu, Jingpeng Li

**Affiliations:** 1 Department of Basic Course Teaching, Shandong Agriculture and Engineering University, Jinan, Shandong, China; 2 School of Information Engineering, Shandong Agriculture and Engineering University, Jinan, Shandong, China; 3 Engineer, Inspur Software Group Co., Ltd., Jinan, Shandong, China; Mae Fah Luang University, THAILAND

## Abstract

Because thermal infrared sport targets have rich and complex semantic information, there is a high coupling between different types of features. In view of these limitations, we propose a Non-Glodal decoupled Attention, namely,local U-shaped attention decoupling network (LUANets), which aims to decompose the coupling relationship of different sport target features in thermal infrared images and establish effective spatial dependence between them. This method takes the captured multi-scale initial features according to different levels and inputs them into the local decoupling module with U-shaped attention structure to realize the decomposition of semantic details. At the same time, considering the correlation between different targets, in the process of feature decomposition, using prior knowledge as guiding information many times to establish effective spatial dependence. Secondly, we design a two-way cross-aggregation FPN module to cross-aggregate information flows in the front and back directions to achieve feature interaction while further reducing the coupling between different types of features. The evaluation results on data such as TIIs,SportFCs and FLIR show that the LUANets method we proposed has achieved the best detection performance, with mAP of 68.72%,59.51% and 65.29%, respectively.

## Introduction

With the development of deep learning technology and infrared sensor technology, object detection tasks based on thermal infrared images have received extensive attention [1]. Thermal infrared images can not only display human body thermal energy information but can also have strong anti-interference ability. For example, in environments where there is not enough lighting during the night or in low-light indoor settings, it has clear advantages compared to RGB optical images. Although deep learning methods are widely used in object detection tasks, there are still many challenges in using thermal infrared images for object detection [2]. First, the existing thermal infrared image detection tasks mostly focus on the detection of a single category. For indoor sports and sports images that contain different categories and large-scale changes, there are fewer relevant studies and low detection precision. In addition, compared to RGB high-resolution optical images, thermal infrared images have lower resolution and contrast. Therefore, how to improve the object detection precision of thermal infrared images has become an important research topic.

Target detection in the early stage mainly uses hand-made feature [3–5], that is, manual participation in feature design and screening. These methods have low precision and are labor-intensive, time-consuming, and costly. In addition, the environmental requirements are relatively high; that is, the application requirements need to be met under specific environmental conditions, which results in most of these methods being only suitable for limited environments. On the other hand, with the development of imaging technology and deep learning technology, deep convolutional neural networks are widely used in many fields, such as image segmentation, recognition and retrieval. Inspired by this, many researchers apply CNNs to target detection tasks but mainly focus on detecting optical image targets. For the target detection of RGB optical images, most of them use VGG, ResNet and DenseNet [6–8] to realize the local feature extraction of the target object. Compared with RGB images, the target structure, content, or feature information in thermal infrared images is more complicated. If only a feature extractor with a single structure is used to achieve the feature capture, the requirements needed to meet the application precision are difficult. In contrast, effective multiscale information is more helpful for the localization and detection of thermal infrared targets. For example, the two-stage fast region convolution (FasterR-CNN) [9–11] has been successfully applied to thermal infrared images to achieve accurate detection of target objects. Although these methods improve the detection precision to a certain extent, in the feature extraction stage, multiple sets of irrelevant redundant information are used, and the rich underlying semantic details are ignored, resulting in low detection precision. However, to solve these limitations, many researchers embed the attention mechanism into the feature extraction stage to achieve refined processing of semantic details, that is, to reduce the use of redundant information and to obtain richer multiscale representations. However, these two-stage methods inevitably increase the feature dimension, that is, reduce the operating efficiency of the model. Therefore, to better balance the detection efficiency and precision, some researchers [12] use a single-stage detection method to achieve accurate detection of infrared targets; that is, dense candidate frames (anchor frames) are generated on feature maps of different layers and scales. The network can obtain effective details and multiscale representations without significantly increasing the computational complexity of the network.

Although the above methods have achieved good performance in object detection tasks in thermal infrared images, during the feature extraction stage, only the high-level semantics of the object are considered, and the representation capabilities of middle- and low-level features are ignored. At the same time, thermal infrared images are not considered. The spatial relationship between objects and the failure to effectively resolve the complementarity between multiscale features results in the loss of detailed information in the detection process. In addition, low-level information such as the physical appearance of the object contains rich detailed semantics. Therefore, in light of these limitations, we propose a local U-shaped attention decoupling network (LUANets) for sports objects in thermal infrared images. First, we use Res2Net-DCN to capture the initial features of the object in the thermal infrared image, that is, the basic multiscale prior features. Second, these prior features are input into the multilevel nonglobal decoupling module to model the local and global spatial relationships of the object and establish more effective long-term dependencies. Finally, to compensate for the lack of single-scale feature representation and the lack of interactivity of features at different levels, a bidirectional cross-aggregation strategy is used to enhance interaction, and at the same time, different features form an effective complement to improve the detection precision of objects in thermal infrared images.

The main contributions of this article are as follows:

- We propose a local U-shaped attention decoupling network (LUANets) for thermal infrared image object detection. The object is described in detail from three levels of low, medium and high, and effective spatial relationships and long-term dependencies are established. Meanwhile, the interaction between features of different layers is realized to form more robust multiscale information. In addition, a priori low-level semantics have been used many times to further strengthen the representation of multiscale features, thereby improving the network’s prediction performance for objects of different scales.
- In the local u-shape attention decoupling module, the squeeze excitation and global attention network are embedded in U-shaped components to decouple the output of different scales and use the pooling operation of different scales to combine coarse features and fine features. In this way, each decoupling level contains detailed semantics of different scales. At the same time, the computational complexity of the model is reduced.
- Using a cross-aggregation strategy, the output features of the multilevel decoupling module are embedded in the bidirectional FPN, and local and global semantic interactions are realized from both the positive and negative directions. This makes the detection framework more sensitive to objects of different scales. Further strengthening the spatial relationship between different goals. The final experimental results show that the proposed detection framework achieves the best performance on baseline data such as TIIs and FLIR.

The rest of this article is organized as follows: In Chapter 2, we will elaborate on the research work related to this article. Chapter 3 introduces the proposed detection framework in detail. The fourth chapter gives the corresponding experimental results, as well as a discussion and analysis. Conclusions and prospects will be described in Chapter.

## Related work

With the successful application of deep learning technology in image segmentation, recognition and many other fields, deep learning technology has also been widely used in target detection tasks. Normally, this method is divided into one-stage and two-stage detection networks. Among them, Faster R-CNN and Cascade R-CNN [13, 14], as classic two-stage detection algorithms, have been applied to many detection tasks with good detection performance. For example, Galarza-Bravo and Flores-Calero [15] combined two regional proposal networks, RPNCD and RPNLD, with FasterR-CNN to improve the detection precision of distant pedestrians and developed a multichannel system that focuses on the detection of distant pedestrians. The standard framework realizes the detection of pedestrians accurately. Li and Song [16]. considered the feature differences between thermal infrared images and RGB images, discussed in depth six convolutional networks of different scales, and proposed a light-perception Faster R-CNN detection framework. Similarly, Dai and Hu et al. [17] proposed a novel multitask Faster R-CNN detector to estimate the safe driving distance to improve the precision of driving only, which adjusts the ResNet-50 feature extractor. Realize distance evaluation while improving feature characterization capabilities. Cascade R-CNN relies on powerful feature captures and fusion capabilities to achieve the task of target detection in thermal infrared images. For example, Wang and Li. [18], considering the poor anti-interference of RGB optical images and the difficulty of traditional convolutional neural networks in capturing the deep abstract information of the target, proposed a high-performance, strong anti-interference thermal infrared star load detection framework, which mainly consists of a cascade RCNN network. Although these two types of methods are superior to traditional detection methods in terms of precision, during the feature extraction stage, it is easy to ignore the detailed semantics of the target. At the same time, the feature extraction ability is poor; that is, the distinguishing features of the target cannot be extracted well. In addition, this type of two-stage detection method requires more computational costs, resulting in low detection efficiency. Therefore, some fast and accurate single-stage detection algorithms are applied. For example, Ivašić-Kos and Krišto. [19] used YOLO to detect pedestrians in thermal infrared video. Li. [20] used YOLO-v3 to detect pedestrians in thermal infrared images at night to compensate for the shortcomings of traditional cameras that cannot be used under poor lighting conditions and achieved better detection precision. However, to further improve the precision of pedestrian detection, Kannadaguli Pdent et al. [21] used the improved YOLO-v4 for pedestrian detection in UAV and thermal infrared images. Feng and Wang. [22] proposed an SSD thermal infrared pedestrian detection algorithm based on migration learning and extended the thermal infrared dataset. Geng et al. [23] combined the migration method to improve yolo-v3 and finally realized the detection of pedestrians in thermal infrared images. These single-stage algorithms have improved the detection efficiency to a certain extent, but the detection precision is open to question. Therefore, under the condition that the detection efficiency is within the receiving range, to further improve the feature extraction and characterization capabilities of the network, many researchers have proposed strategies such as multifeatured fusion, multiscale deep extraction and attention aggregation.

Feature fusion and multiscale strategies can describe and represent targets from multiple perspectives, and different features can complement each other. Attention aims to focus on key positions, strengthen key information, and weaken the ability to express irrelevant information. For example, Dai X and Yuan X et al. [24] proposed a new TIRNet thermal infrared target detection method, that is, using a lightweight multiscale VGG to replace deep CNN, and to capture more robust discriminative features, the introduction of a residual difference branch to achieve the optimization of the feature extractor. To alleviate the error caused by the external environment on the detection of optical image targets, Shao and Li et al. [25] proposed a detection algorithm that uses two types of features together; that is, the use of multitask cascaded CNN optical features and multiscale CNN to capture heat infrared optical flow information realizes the complementarity between features, thereby improving the precision of pedestrian detection. Tu and Li et al. [26] proposed a multi-interactive dual-translation encoder to interact with multilevel features and global context information and finally achieved the precise detection of RGB-thermal infrared salient targets. Hu and Gao et al. [27] proposed a spatiotemporal segmentation model based on a multidimensional feature fusion structure for automatic thermal infrared image defect detection. This method enables local interaction between adjacent pixels and adaptively recorrects the feature map., and improve the characterization ability of features. Considering the limitation of single-modal salient target detection performance, Wang and Song et al. [28] proposed a new cross-guided fusion network (CGFNet) for RGB-T salient target detection, which realizes the crossover between features. Modal interaction. Although this type of method improves the expression of features to a certain extent and at the same time improves the precision of target detection, it causes the use of redundant information in the process of feature fusion and multiscale information capture, ignoring the extraction of detailed semantics. Therefore, the attention mechanism is widely used in thermal infrared target detection tasks. For example, Munir and Azam et al. [29] considered that multispectral images can provide complementary information for thermal infrared images and proposed an attention-guided feature fusion method to achieve accurate detection of pedestrians in thermal infrared images. Zhu and Dou et al. [30] proposed a multiscale channel attention image fusion method to achieve the fusion of visible light and thermal infrared images while improving the precision of target detection. Considering that the poor resolution of thermal infrared images limits the feature extraction capabilities of the network, Zhang and Xu et al. [31] proposed a new backbone network, Deep-IRobject, to achieve target feature extraction and fusion. The backbone of the network uses channels. The attention block and the location attention block extract the interdependence between the channel dimension and the location dimension, respectively, and obtain the semantic information of the channel and the location. Considering that traditional target detection methods usually require a large amount of training data, Qi Fan, Wei Zhuo et al. [32] proposed a novel small number of shooting target detection networks, which aim to detect invisible targets through a small number of samples. The core of this method is to combine the attention mechanism with RPN, including multiple relational detectors and comparative training strategies, using the similarity between a small number of shot support sets and query sets to detect new targets, and effectively suppress false results in the background target. Ramin Nabati, Hairong Qi [33] and others proposed an intermediate fusion method for 3D target detection using radar and camera data. First, a center point detection network is used to detect objects by identifying the center point on the image. Then, a truncated cone-based method is used to associate radar detection with the target center point, which solves the key data association problem. Yizhou Wang, Zhongyu [34] Jiang and others proposed a deep radar target detection network (RODNet) to effectively detect targets, purely from carefully processed radar frequency data, to achieve target positioning in the form of range-azimuth frequency heatmaps (RAMaps).

The abovementioned methods obtain better feature expression to a certain extent but only consider the high-level semantics of the target and ignore the rich details contained in the low-level physical appearance. At the same time, they cannot establish effective features between different scales, distinct levels, relationship and long-term dependence. In addition, these methods mainly focus on target detection in natural RGB images, while thermal infrared detection methods mostly focus on pedestrian detection, with only a few studies on target detection tasks for sports. Therefore, to solve these limitations, we propose a novel nonglobally decoupled attention network for sports target detection in thermal infrared images.

## Our proposed framework

In this section, we first focus on the basic process of the proposed local U-shaped attention decoupling network (LUANets) detection framework. Second, this section describes in detail the important components of the initial prior feature extraction module, the lobal u-shape attention decoupling module (LUADM) and the bidirectional cross-aggregation FPN (BCFPN).

### Basic overview

**Fig 1:**
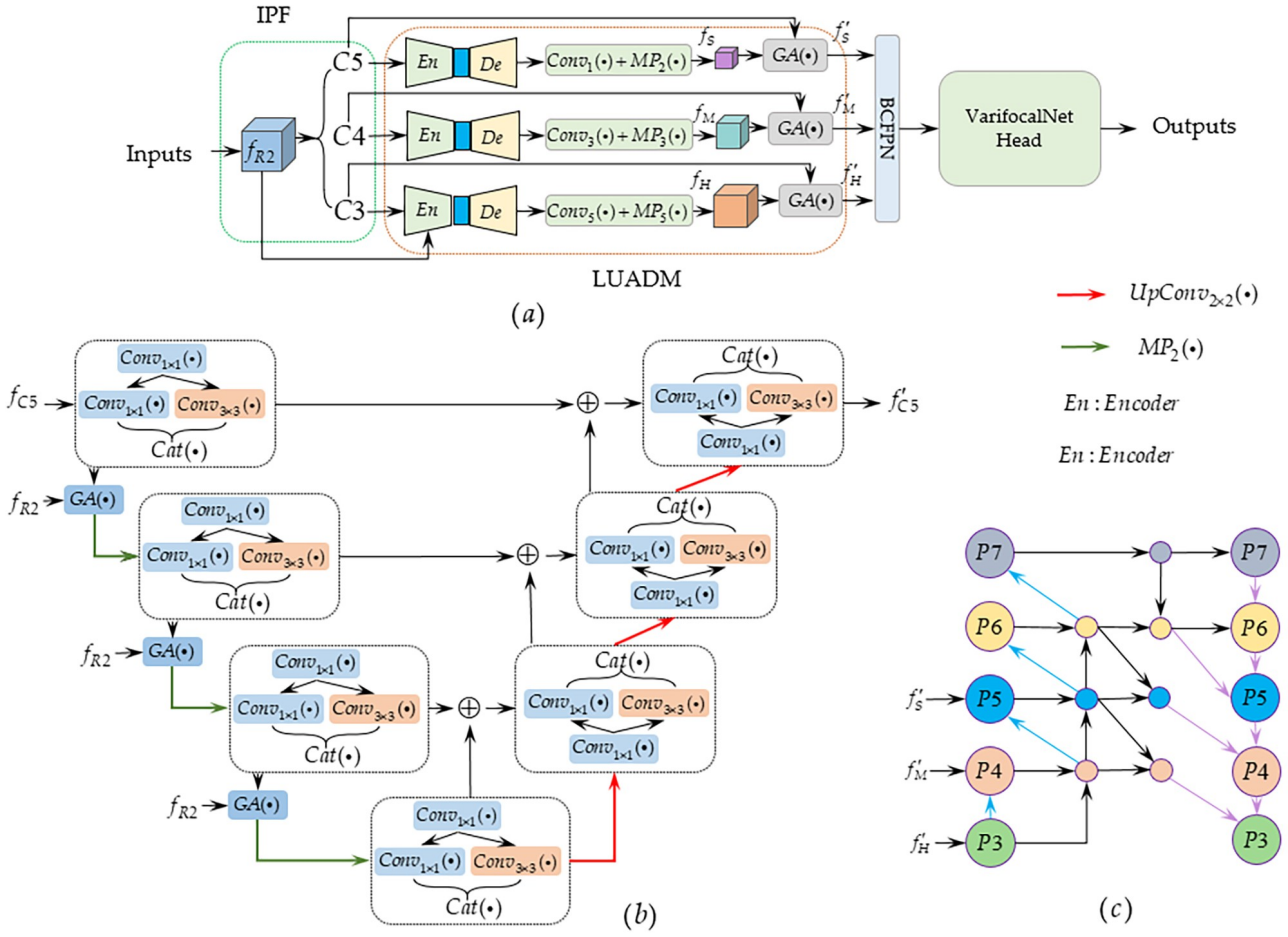
(a) The overall structure of the LUANets detection framework. (b) Represents the LUADM of *C*5. (c) The bidirectional cross-aggregation FPN (BCFPN) module. *f*_*R*2_ represents the features extracted by Res2Net-101 [35]. *C*3, *C*4, *C*5 respectively represent the convolution operation of different hole coefficients, and the output feature can be defined as *f*_*C*3_, *f*_*C*4_, *f*_*C*5_. *EN*, *De* represents the encoder and decoder, respectively. *GA*(⋯) represents the global attention operation. *f*_*s*_, *f*_*M*_, *f*_*H*_ represent different levels of feature information, such as low, middle, and high. *BCFPN* represents the bidirectional cross-aggregation FPN module. *Conv*_1 × 1_ means that the convolution kernel is 1 × 1, *RELU* + *BN* and other operations. represents the feature map connection. *MP*_2,3,5_(⋅) represents the maximum pooling operation of separate scales. ⊕ means skip connection. {*P*_5_, ⋯, *P*_7_} represents a pyramid structure of different levels.

Deep convolution and feature pyramid networks have achieved good performance in many detection tasks by virtue of their powerful feature capture capabilities. However, it is not sensitive to the detailed information of thermal infrared objects, and at the same time, it is easy to reuse redundant information, resulting in poor detection precision and efficiency. In addition, considering the low resolution of thermal infrared images, poor texture information, and complex content, as well as the lack of prior details in the feature capture process, weak feature identification capabilities are achieved. We propose a local U-shaped attention decoupling detection framework (LUANets), which effectively aggregates multilevel information and simultaneously improves the modeling of global context dependencies.It is worth noting that multiple U-shaped decoupling blocks and attention work together to form a multi-scale guided multi-level U-shaped decoupling network (MGUN), which can obtain multi-scale features and realize the fusion of different levels of feature information. The overall structure of LUANets is shown in Fig 1.

### IPF module

Considering that prior knowledge contains rich low-level semantics, it is helpful for the description of semantic details, and the deep residual network is used to extract multiscale [36] features of sports targets in thermal infrared images. However, when the information flow is transmitted in the residual layer, it can easily cause a large amount of redundant information. At the same time, to obtain better multiscale prior features, Res2Net-101 is combined with hole convolution (DCN), which is the network’s block feature. The convolution operations in the third, fourth, and fifth residual blocks are replaced with hole convolutions to form better multiscale features, such as *C*3, *C*4, *C*5 in Fig 1(a), which will output the feature map defined as *f*_*C*3_, *f*_*C*4_, *f*_*C*5_. However, considering the importance of prior knowledge, we define the output feature before the hole convolution operation as the initial prior feature *f*_*R*2_.

### LUADM module

Fig 1 (b) is a low-level decoupling module, which is mainly composed of a U-shaped network of encoder and decoder. In the encoder, we use and to decompose and fuse low-level features to form a more refined multiscale representation. Second, the initial prior features and this information are used together as the input of the global attention (GA) component to achieve encoding, and on the decoder, the skip link is used to perform up sampling operations at the same time to capture the distinguished detailed information and focus on the representation of relevant characteristics. In addition, each encoding and decoding block contains an extrusion layer and an excitation layer. To reduce the number of parameters of the model, a smaller network is created.

#### Encoder module

Using the squeeze excitation layer in this module can not only improve the ability to capture multiscale features but also significantly reduce the number of learnable parameters and computational complexity. Second, global attention is used to map the decomposed and fused multiscale features with prior knowledge. which reduces the use of redundant information while using prior knowledge *f*_*R*2_. Assuming that the feature inputs are $X_{ml}^{\left(l\right)}$ the output of the extrusion block is as shown in the following equation:

$$\begin{array}{c} o_{sq}^{\left(l\right)}=Conv_{1\times 1}\left(X_{ml}^{\left(l\right)}\right) \end{array}$$ (1)

where *Conv*_1×1_(⋅) represents the convolution operation of $1\times 1.o_{sq}^{\left(l\right)}$ represents the output of the *l*^*th*^ squeeze layer.

Second, the squeezed output is passed to the parallel excitation layer of two different convolution kernels to realize the feature map decomposition. In addition, the output features of the parallel excitation layer are spliced. This process is defined as the following equation:

$$\begin{array}{c} o_{ex}^{\left(l\right)}=Cat\left[Conv_{1\times 1}\left(o_{sq}^{\left(l\right)}\right),Conv_{3\times 3}\left(o_{sq}^{\left(l\right)}\right)\right] \end{array}$$ (2)

where $o_{ex}^{\left(l\right)}$ represents the splicing feature of the *l*^*th*^ parallel excitation layer. *Conv*_3×3_(⋅) represents the convolution operation of 3 × 3.

Finally, we use the global attention module (GA) to refine the prior knowledge *f*_*R*2_ and the output of the parallel excitation layer $o_{ex}^{\left(l\right)}$ to refine and filter irrelevant redundant information. In addition, the maximum pooling operation of 2×2 is used to map this multiscale information to a unified low-level space to build spatial dependencies. These processes are shown in the following equations:

$$\{\begin{array}{l} o_{ga}^{\left(l\right)}=GA(o_{ex}^{\left(l\right)},f_{R2})\\ o_{ml}^{\left(l\right)}=MaxP_{2\times 2}(o_{ga}^{\left(l\right)}) \end{array}$$ (3)

where $o_{ml}^{\left(l\right)}$ represents the multiscale spatial semantics after the maximum pooling operation. represents the decomposition and fusion features obtained through the global attention module. *MaxP*_2×2_ represents the maximum pooling operation of 2 × 2.

#### Decoding module

Where to the structure of the encoding module, this decoder uses a transposed squeeze excitation operation to reduce the number of model parameters, and its main component is an up-sampling unit. Each up-sampling unit is composed of a set of transposed extruded excitation layers, the transposed extruded layer is composed of transposed convolution of 1 × 1 and the transposed convolution of 1 × 1. The output is transferred to the excitation convolution layer of two different convolution kernels while splice aggregating the output transposed squeeze excitation module. The difference from the encoding block is that we simply add these transposed outputs and the corresponding encoded output, together as a gate control for the information to enable the decoder to include certain context semantics. At the same time, the skip connection helps to learn from the encoder. This encoder extracts the richest details of the semantics. In addition, for the effective transmission of information flow and the communication between features of different scales, the transposed output is reinput to the lower decoding block, and the up-sampling pooling operation is performed to obtain the most effective key information. Assuming that the output of the transposed splicing output in the decoder is $o_{de}^{\left(l\right)}$, the decoding input and skip connection are as shown in the following equation:

$$\{\begin{array}{l} o_{skip}^{\left(l\right)}=Cat[o_{de}^{\left(l\right)},o_{en}^{\left(l\right)}]GA(o_{ex}^{\left(l\right)},f_{R2})\\ o_{up}^{\left(l\right)}=MaxP_{2\times 2}(o_{de}^{\left(l\right)})\\ o_{de_{i}}^{\left(l+1\right)}=Cat[o_{skip}^{\left(l\right)},o_{up}^{\left(l\right)}] \end{array}$$ (4)

Where, $o_{up}^{\left(l\right)}$ indicated the up-sampling output of up sampling layers. $o_{de}^{\left(l\right)}$ represents the output of the decoded block. $o_{skip}^{\left(l\right)}$ represents the output of the *l* skip connection. $o_{en}^{\left(l\right)}$ represents the output of the *l* corresponding encoding module. $o_{de_{i}}^{\left(l+1\right)}$ represents the input of the *l* + 1 decoding module.

However, to capture more effective details, we have performed a partial decomposition of the output of the three different levels. That is, three U-shaped networks with similar structures are used to decouple and integrate *C*3, *C*4, *C*5 to realize the modeling of spatial relationships and long-term dependence. In addition, to better obtain multilevel semantic details and establish global associations, we use different scales of convolution and pooling operations to compress and expand the decoupling features of the U-shaped module, that is, the high-level (*C*3) feature large-scale convolution and pooling operations to obtain the final high-level semantic *f*_*H*_, and the middle layer uses 3 × 3 convolution pooling to obtain the extended middle layer semantics. Second, the global attention module is used to realize the detailed processing of different layers of semantic information. The specific operation is as follows:

$$\{\begin{array}{l} f_{H}=MaxP_{5\times 5}(o_{de}^{C3})\\ f_{H}^{\prime }=GA(f_{C3},f_{H}) \end{array}$$ (5)

where *MaxP*_5×5_(⋅) represents the pooling operation of 5 × 5. represents the output of the U-type module after global decoupling. For *C*3, *C*4 and *C*5 are consistent in theory.

### BCFPN module

There is a strong correlation and dependence when the complementary relationship between the features of different levels at the same time. We designed a two-way feature pyramid network with a cross-aggregation strategy, which makes the pyramid layers of different structures have bottom-up forward global semantics and aggregates the reverse details from top to bottom. However, when the traditional two-way pyramid structure aggregates information forward and backward, the information flow often comes from the upper layer and the current layer, which leads to a weak long-term dependence between the target features and poor interaction between low-, medium-, and high-level features. Therefore, to achieve the direct interaction and dependence of separate layer features, we transfer the information flow across layers when each pyramid level has semantic details such as large, medium and small at the same time. Assume that the forward and backward output features of the pyramid layer are $\overset{↔}{f_{P3}},\overset{↔}{f_{P4}},\overset{↔}{f_{P5}}$ respectively.

#### For the positive feature pyramid

*For the forward feature pyramid*. For the *P*5 layer in the forward feature pyramid network (FPN) [37, 38], we first stitch the forward output features of *P*4 with the forward output features of the *P*3 fusion and use the convolution of 1 × 1 to map and transform the input *P*5 layer, and for the *P*6 and *P*7 layers, the pyramid information of the first 6 layers will be merged in turn. This is conducive to the spread of multiscale features within the pyramid and to strengthening the correlation between features at different levels. The fusion process of different layers is shown in the following equation:

$$\{\begin{array}{l} \vec{f_{P5}^{\prime }}=Conv_{1\times 1}(Cat[\vec{f_{P3}},\vec{f_{P4}}])\\ \vec{f_{P6}^{\prime }}=Conv_{1\times 1}(Cat[Cat[\vec{f_{P3}},\vec{f_{P4}}],\vec{f_{P5}}])\\ \vec{f_{{P7}^{\prime }}}=Conv_{1\times 1}(Cat[Cat[Cat[\vec{f_{P3}},\vec{f_{P4}}],\vec{f_{P5}}],\vec{f_{P6}}]) \end{array}$$ (6)

Where, $\vec{f_{P5}^{\prime }},\vec{f_{P6}^{\prime }},\vec{f_{P7}^{\prime }}$ represent the input features of the forward pyramid *P*5, *P*6, *P*7. *Cat*[⋅, ⋅] represents a simple splicing operation. With these operations, we can achieve cross-layer interaction of low, medium, and high-level semantic information and improve the use of detailed information, that is, without increasing the number of model parameters, further reusing positive semantics.

To capture the reverse global semantics of the target features and improve the representation ability of contextual semantics, we also use cross-aggregation in the process of backpropagation. For the input information of the reverse layer, we will perform an up sampling operation on the fusion features of the forward *P*3 layer and the fusion of the *P*3, *P*4 layer, that is, the reverse *P*3. The input of the layer is shown in the equation.

$$\{\begin{array}{l} \overset{\leftarrow }{f_{P3}^{\prime }}=UpConv_{1\times 1}([Cat[\vec{f_{P5}^{\prime }},\vec{f_{P5}}],\overset{\leftarrow }{P4}])\\ \overset{\leftarrow }{f_{P4}^{\prime }}=UpConv_{1\times 1}([Cat[\vec{f_{P6}^{\prime }},\vec{f_{P6}}],\overset{\leftarrow }{P5}])\\ \overset{\leftarrow }{f_{P5}^{\prime }}=UpConv_{1\times 1}([Cat[\vec{f_{P7}^{\prime }},\vec{f_{P7}}],\overset{\leftarrow }{P6}]) \end{array}$$ (7)

Where, $\overset{\leftarrow }{f_{P3}^{\prime }},\overset{\leftarrow }{f_{P4}^{\prime }},\overset{\leftarrow }{f_{P5}^{\prime }}$ respectively represent the input feature maps of reverse *P*3, *P*4, *P*5. $\overset{\leftarrow }{f_{P6}^{\prime }}$ denote reverse *P*6 output feature map.

In short, we use nonglobal decoupling modules and two-way cross-aggregation FPN to help the use of low-level details, establish effective associations between features of different scales, and use cross-aggregation strategies to achieve multilevel feature interaction within the pyramid. To a certain extent, the multilevel approach has realized the complementarity between different levels of features and established an effective dependency relationship. Finally, to achieve accurate detection of sports targets in thermal infrared images, VarifocalNet Head [39] is used for detection.

## Experimental results

In this section, we analyze and discuss the proposed detection framework in two ways, qualitatively and quantitatively, and demonstrate the effectiveness of the framework. Next, the comparison with other advanced methods, the influence of different components on the overall performance of the proposed framework, and the related ablation research and visualization discussion and analysis will be elaborated.

### Datasets preparation

To verify the effectiveness of the proposed detection framework, this paper uses three open baseline datasets, among which a series of operations, such as cropping and box conversion, are required for thermal infrared images.

#### TIIs

The data mainly include 1500 thermal infrared images of football sports in the field, and the images are cropped to size. Each image contains 8 football players, that is, each image includes 8 different categories. To ensure the consistency of the experiment, we will randomly select 900 thermal infrared images for model training, and the remaining 600 will be used for test verification. To avoid the fitting phenomenon of the detection frame, a variety of data enhancement methods, such as random rotation, color transformation and Gaussian blur, are introduced. It is worth noting that for thermal infrared images, we use a rotation every 30 degrees to increase the number of training images. The number of thermal infrared images used for training through the data enhancement strategy is 8742, and the test samples remain unchanged.

#### SportFCs

The data mainly include 3000 RGB visible light images, of which 1874 images include the football category, and 1126 images include the cricket category. At the same time, to ensure the fairness of the experiment, we randomly selected 60% of the images of each category for model training, and the remaining 40% was used for test demonstration.

#### FLIR

[12] The dataset contains a total of 14452 infrared images, of which 10228 are from multiple short videos and 4224 are from a 144 s video. All videos are from streets and highways. The sampling rate of most pictures is two frames per second, and the frame rate of video is 30 frames per second; in a few environments, the sampling rate is 1 frame per second. To ensure the smooth progress of the experiment, we randomly selected 8862 of them for training. The number of verification samples was 1366, and the rest were test samples. It is worth noting that these samples all include five categories: people, bicycles, cars, dogs, and others.

### Experimental environment and training settings

#### Training parameters

We use stochastic gradient descent (SGD) to adjust and optimize the proposed detection framework, where the initial learning rate is 0.0005, the batch size is 2, the weight decay rate is 0.0001, 1920×480 and 480×800 the momentum is 0.9. To effectively utilize the multiscale features of the object in the image, since the thermal infrared image is of a fixed size, we use two scales: and for training, and the number of iterations is 210. For the RGB dataset, we iterate only 150 times but use 224×480, 480×800,800×800 and 800×512. Scale describes the multiscale information. In the proposed model framework, we use Res2Net+DCN as the backbone network for multiscale feature extraction, and the parameters of the backbone network are pretrained on the ImageNet dataset. In the local decoupling module, each nonglobal embedded U-shaped component includes 3 encoding blocks, 1 conversion block and 3 decoding blocks, and the output feature maps are 256, 512 and 1024. Regarding bidirectional cross-aggregation, FPN contains three scales: large, medium and small. The prediction stage is similar in structure to VFNet but only contains three scales; that is, VarifocalNet Head only effectively aggregates the feature information of the three scales, and the others remain the same. It is worth noting that in the quantitative demonstration stage, the training set is 10%, 20%, 30% and 40%.

#### Training environment

We use Torch 1.7.0+cu110+Python 3.7.3 to build the detection framework and implement training and prediction on two RTX 2080Ti GPUs. It is worth noting that all comparative experiments are tested and verified on this GPU, and deep learning libraries such as opencv 4.5.3.46 and Numpy are used.

### Evaluation metrics

To ensure the validity and consistency of the experiment, we use the average precision (mAP) and recall rate (R) to evaluate the proposed detection framework and comparison algorithm. The calculation process is shown in the equation.

$$\{\begin{array}{l} P=\frac{TP}{TP+FP}\\ mAP=\frac{P}{N}\\ R=\frac{TP}{TP+FN} \end{array}$$ (8)

where TP is the number of true positives, FP is the number of false-positives, FN is the number of false negatives, and N indicates the number of classes. The higher the P value, the better the test results.

### Experimental results and analysis

To demonstrate the effectiveness of the proposed detection framework, this chapter gives relevant experimental results and analyzes and discusses the proposed framework and other detection algorithms. It is worth noting that all algorithms use baseline data such as FLIR, TIIs and SportFCs as experimental samples. The experimental results of all detection methods are shown in Table 1.

**Table 1:** Experimental results of different detection methods.

models	Backbone	TIIs		SportFCs		FLIR	
		R	mAP	R	mAP	R	mAP
FasterR-CNN	X-101-64-4d-FPN	66.85	65.08	58.90	55.85	62.33	60.15
CascadeR-CNN	x101-64-4d-FPN	67.86	66.91	59.26	58.19	64.67	62.09
DETR	R50-8-2	64.02	62.84	55.96	53.07	59.99	56.84
Yolof	R50-c5-8-8	66.75	64.56	57.87	55.08	61.79	58.15
AutoAssign	R50-FPN-8-2	65.31	63.96	56.72	54.61	60.32	57.62
PVTv2	pvtv2-b2-FPN	68.56	67.93	59.45	58.63	65.55	62.97
VFNet	x101-64-4d-FPN-C3-C5	68.47	67.84	59.39	58.23	65.18	62.36
**LUANets**	**Res2Net101DCN-MGUN-BCFPN**	**69.91**	**68.72**	**62.04**	**59.51**	**69.06**	**65.29**

LUANets represents our proposed detection framework. MGUN represents our proposed local embedded U-shaped module. BCFPN represents our proposed bidirectional cross-aggregation FPN.

According to Table 1, we can draw the following conclusions:

1. Compared with other detection algorithms, our non-GDANet detection framework achieves the best results on these three baseline datasets, namely, mAP is 68.72%, 59.51% and 65.29%, respectively. There may be two reasons: On the one hand, we embed a global attention network in the MGUN module to optimize the backbone network to capture multiscale information and delete irrelevant redundant information. At the same time, the encoding and decoding paths further capture the local dependencies of the object to be detected and the key global context semantics. On the other hand, the bidirectional cross-aggregation FPN (BCFPN) strategy is adopted to strengthen the interaction between three different scales of information, large, medium and small, so that it can produce a certain complement in the feature space, forming a more robust pyramid structure information. At the same time, the aggregation of multiscale information in two directions, positive and negative, is helpful to the modeling of long-term relationships, thereby forming a robust long-term dependency relationship. It is worth noting that we use a multiscale incentive squeeze module in the U-shaped component and integrate the initial features captured by the backbone network, that is, prior knowledge, which is helpful for the representation of low-level semantics such as physical appearance.
2. Compared with the DETR detection method, Faster R-CNN and Cascade R-CNN have achieved better detection results; that is, the mAP value is increased by 2.24% and 4.07% on the TII dataset, respectively, and a deeper residual is used. As the backbone network, the poor network can extract more abstract information. YOLOf adds a hollow convolution (c5) to the R50 backbone network to obtain sparse feature information, which improves the representation of multiscale features, thereby improving the final detection precision. AutoAssign optimizes the pyramid structure of the multiscale information captured by R50, which makes the multiscale information more refined, improves the ability to express detailed information, and increases the amount of computing on the backbone network, which reduces the efficiency of the model.
3. Compared with FasterR-CNN and CascadeR-CNN, the VFNet detection framework has achieved a certain competitive advantage on FLIR baseline datasets, namely, the mAP value is increased by 0.27% and 4.07% on the TII dataset, respectively. It mainly features the third- and fifth-layer output of ResNxt-101 fusion, that is, adding a hollow convolution kernel (c3-c5) to obtain effective local fusion features, but the precision improvement effect is not very obvious, which shows that simply increasing the expression of local information is not enough to achieve the precision of multiscale object detection. PVTv2 shows a strong competitive advantage. The possible explanation is that overlapping blocks with zero-padding embedded in the framework can better extract the location information of the object and use a complex linear attention layer to filter the use of redundant information. At the same time, the characterization ability of key information is strengthened, thereby capturing better detection precision.

### Ablation study

In this section, we will describe in detail the detection performance of separate components, as well as the visualization effects of the MGUN and BCFPN modules.

#### Detection performance of different components

To demonstrate the influence of each component on the overall performance of the proposed LUANets detection framework, we conducted experimental tests on thermal infrared datasets TTIs and gave the experimental effects and evaluation analysis of different components. The specific experimental results are shown in Table 2.

**Table 2:** Detection results of the different components.

Model	Backbone	R (%)	mAP (%)
**IFE**	Resnet-50	64.40	62.97
	ResNet-101	65.59	63.48
	ResNet-50-DCN	65.99	63.76
	ResNet-101-DCN	66.52	65.17
**MLF**	MGUN	66.79	65.44
	BCFPN	67.15	65.74
**IS**	*MFCN*_1,2_ + *BCFPN*	67.58	66.27
	*MFCN*_1,3_ + *BCFPN*	68.07	66.55
	*MFCN*_2,3_ + *BCFPN*	66.98	65.82
	MGUN+FPN	68.44	66.96
	MGUN+PCN	68.87	67.24
**LUANets**	NoMGUN	65.12	63.88
	NoBCFPN	62.99	60.01
	NoDCN	68.33	66.95
	MGUN+BCFPN	69.91	68.72

MGUN represents our multilevel local embedding U-shaped model. BCFPN represents our proposed bidirectional cross-aggregation FPN module. FPN stands for Feature Pyramid Network. MLF stands for a multilevel feature extractor, which mainly includes low-level, middle-level and high-level semantic information. IS represents the internal structure of the MGUN and BCFPN modules. In IFE, MGUN and BCFPN remain unchanged, and only the initial feature extractor is changed. The initial feature extractor Res2Net-101+DCN in MLF remains unchanged, only changing the representation process of multiple hierarchical information. The IS keeps the original feature extractor unchanged while changing the internal structure of the multilevel information extraction component. For example, *MFCN*_1,2_ means that only low-level and middle-level multiscale information is used. PCN stands for pyramid convolution structure. DCN indicates C3 C5.

From Table 2, we can clearly draw the following conclusions.

1. On these baseline datasets, as the network of the initial feature extractor deepens, the detection precision also increases. For example, on the TTI dataset, ResNet-101 is 0.51% higher than the mAP of ResNet-50. ResNet Compared with ResNet-50+DCN, the R and mAP of Res2Net-101+DCN are increased by 0.53% and 1.41%, respectively. On the FLIP dataset, the R and mAP of ResNet-101+DCN are improved by 0.67% and 1.59%, respectively, compared with ResNet-50+DCN. The main reason is that deeper feature extractors can capture better deep abstract information, which is often discriminative and effectively distinguishes the differences between different types of targets.
2. Compared with the MGUN multilevel information fusion module, BCFPN has achieved a better competitive advantage. For example, on the TTI dataset, mAP increased by 0.30%. This shows that the BCDPN module has better multilevel information fusion capabilities than MGUNs. The possible reason is that we have strengthened the interaction between multiscale information in the positive and negative directions through the two-way cross-aggregation strategy. At the same time, it has effectively improved the representation ability of global and contextual semantic information and highlighted the detailed information of key areas. The reason for the poor performance of the MGUN module may be that when the U-shaped network is used to capture multiscale information at different levels, some details are ignored. At the same time, in the process of fusing multilevel information, multiple levels of redundant information are used to weaken the key information that is characterized, and the aggregation ability of global contextual semantic information is poor.
3. Compared with the *MFCN*_2,3_+ *BCFPN* module, both *MFCN*_1,2_+ *BCFPN* and *MFCN*_1,3_+ *BCFPN* have achieved better detection precision. At the same time, the R and mAP of are increased by 0.49% and 0.22%, respectively, compared with *MFCN*_1,3_+ *BCFPN*. The fusion of low-level and high-level information improves the ability of multiscale features to represent the target object. If only the high-level and middle-level information are modeled, it is easy to lose the rich semantics contained in the low-level information. This makes the detection framework insensitive to detailed information.

It is worth noting that the detection precision of MGUN+PCN is 0.43% and 0.28% higher than that of MGUN+FPN. Because the pyramid convolutional network adds a small-scale convolutional layer to the original FPN, it makes it easier to capture detailed information, and at the same time, it further strengthens the characterization ability of detailed information. Thereby improving the detection precision. In addition, compared with NoMGUN and NoBCFPN, NoDCN has achieved a certain competitive advantage, that is, mAP has increased by 3.07% and 6.94% respectively. This shows that DCN ability to capture multi-scale and multi-level features is limited. At the same time, it can also be clearly seen that NoBCFPN has the worst detection precision, which means that BCFPN helps the network to capture more accurate feature details.

#### Visualization

**Fig 2:**
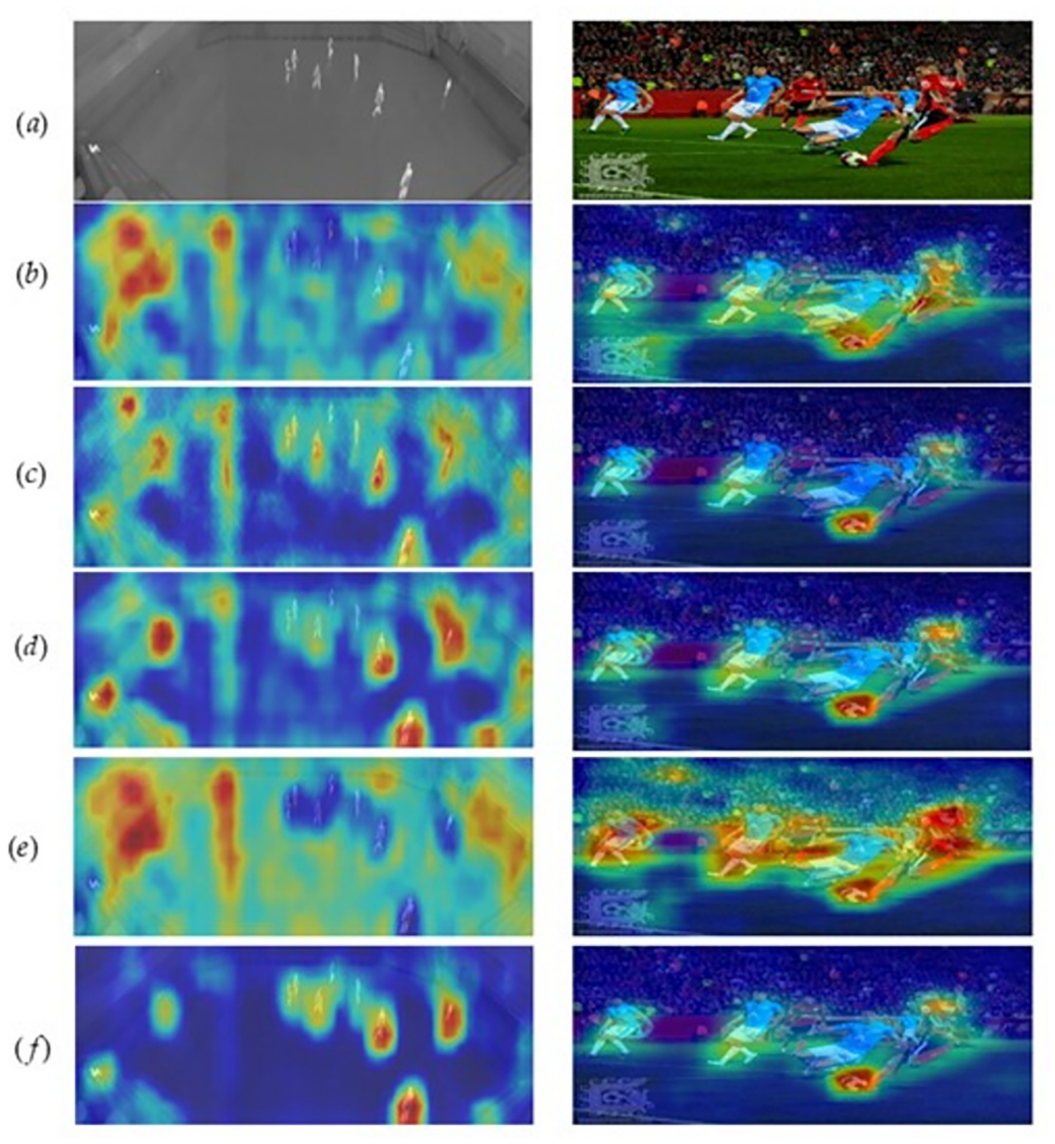
Visualization of different components. (a) represents the original image; (b) represents *MFCN*_2,3_+ *BCFPN*. (c) and (d) represent the visual demonstrations of *MFCN*_1,2_+ *BCFPN* and *MFCN*_1,3_+ *BCFPN*, respectively. (e) represents the BDFPN component. (f) represents the visual presentation of LUANets we mentioned, which includes three levels of detailed information MGUN+BCFPN: low-level, medium-level, and high-level.

To more intuitively illustrate the performance of the MGUN and BCDPN modules in the proposed framework, we give a related visual heatmap. The specific effect is shown in Fig 2. According to Fig 2, we can see.

1. Modeling low-, middle- and high-level information is conducive to the representation of detailed semantics. As shown in Fig 2(f), it can be clearly seen that the focus of the network is more on the object to be detected, which makes the edges or object contours between different types of objects clearer. Simply put, MGUN+BCFPN uses multilevel information to comprehensively describe the object from different angles and levels, uses a bidirectional cross-aggregation strategy to strengthen the interaction between different levels of features, and establishes a robust long-term dependency relationship. It is worth noting that the nonglobal attention layer embedded in the U-shaped module can effectively avoid the use of redundant information and weaken the expression ability of irrelevant information. Therefore, the information flow better retains the key points when passing between layers. Detailed information.
2. Compared with Fig 2(b) and 2(c) *MFCN*_1,2_+ *BCFPN* and Fig 2(d) pay more attention to close to the object to be detected. The possible reason is that these two modules integrate rich low-level semantics. The low-level semantics contain physical appearance information such as the size, distribution, and shape of the object, which enhances the ability to distinguish between different classes and effectively avoids class or class. Increasing gaps.
3. It is also obvious from Fig 2 that the use of the MGUN module helps the framework locate the object; that is, it can better focus on the key information of the object. The attention information in Fig 2(e) is relatively scattered. This may be because the BCFPN module helps to capture global and following information, but the ability to capture local details is poor.

### Sample discussion

To verify the effectiveness of the proposed LUANets detection framework on a small sample dataset, we will randomly select 10%, 20%, 30% and 40% from the SportFC baseline data as training samples and apply them to multiple detection algorithms. Conduct evaluation and demonstration. The experimental results are shown in Table 3.

**Table 3:** Detection results of the different components.

Model	10%	20%	30%	40%
FasterR-CNN	17.43	20.14	25.94	38.78
CascadeR-CNN	18.07	20.51	27.34	40.15
DETR	15.65	17.98	22.56	34.99
Yolof	16.96	20.01	25.38	37.95
AutoAssign	16.31	19.46	24.77	36.88
PVTv2	18.86	20.78	27.42	40.53
VFNet	18.32	20.41	26.99	40.02
LUANets	18.09	21.56	30.19	42.77

MGUN represents our multilevel local embedding U-shaped model. BCFPN represents our proposed bidirectional cross-aggregation FPN module. FPN stands for Feature Pyramid Network. MLF stands for a multilevel feature extractor, which mainly includes low-level, middle-level and high-level semantic information. IS represents the internal structure of the MGUN and BCFPN modules. In IFE, MGUN and BCFPN remain unchanged, and only the initial feature extractor is changed. The initial feature extractor Res2Net-101+DCN in MLF remains unchanged, only changing the representation process of multiple hierarchical information. The IS keeps the original feature extractor unchanged while changing the internal structure of the multilevel information extraction component. For example, *MFCN*_1,2_ means that only low-level and middle-level multiscale information is used. PCN stands for pyramid convolution structure. DCN indicates C3 C5.

According to Table 3, we can draw the following conclusions:

1. For TTIs and FLIP datasets, as the number of training samples increases, the precision of all detection algorithms also increases. A large number of training samples help the model learn more useful details. Among them, on 10% of the training samples, DETR has the lowest detection precision because DETR has a poor ability to capture initial features, and at the same time, a large number of detailed semantics are ignored in the process of information encoding and decoding. PVTv2 is conducive to the capture of low-level semantics and simultaneously strengthens the location information of the object. Therefore, the best detection precision is achieved on small-scale data, that is, the mAP is 18.86%.
2. Compared with small sample data, the increase of training sample data, the increase of detection precision of our proposed LUANets framework is more obvious. For example, on the training samples of 30% and 40%, they are 2.77% and 2.24% higher than PVTv2, respectively. The possible main reason is the use of multilevel local embedding and bidirectional cross-aggregation strategies, which can obtain useful details from large-scale data, or when the data distribution is more complex, it is more conducive to the proposed detection framework capturing multilevel details. Overall, our non-GDANet detection framework can still achieve a certain competitive advantage in small sample data.

**Fig 3:**
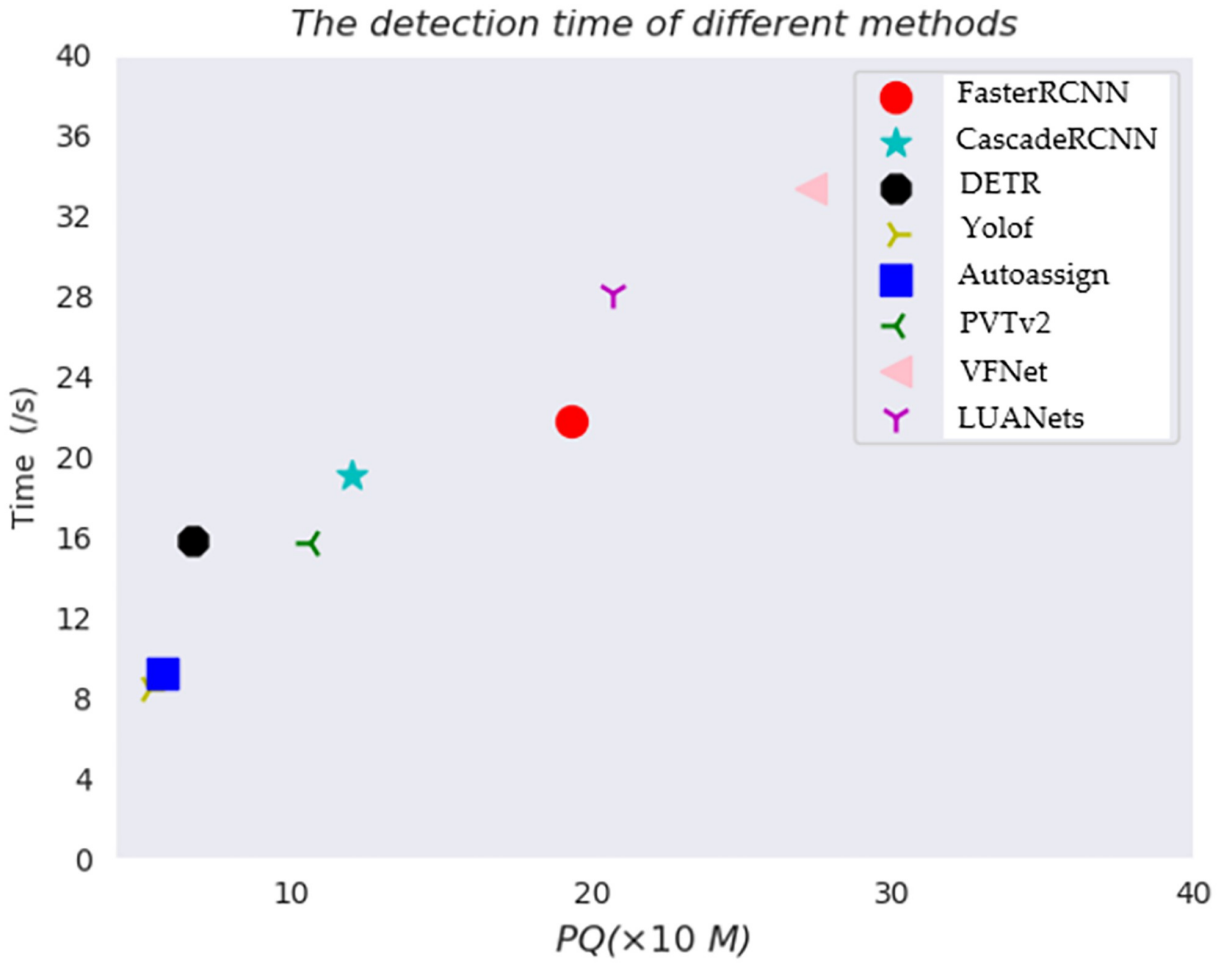
Detection efficiency of the different models. M indicates that the model parameter is megabytes, and s indicates the time it takes for every 100 images to be detected.

To demonstrate that the proposed method has excellent detection efficiency under the condition of ensuring detection precision, we use TIIs as the test sample to give the detection time of 100 images and the parameter quantity (PQ) of each model. The test result is shown in Fig 3.

According to Fig 3, we can clearly see that VFNet has the lowest efficiency in detecting 100 images, and our proposed LUANets has a competitive advantage while maintaining the best precision in the detection efficiency. Compared with VFNet, it is because we compress and crop the captured multiscale information to the three scale ranges of large, medium and small, which saves the capture time of the features in the feature pyramid structure to an extent. Compared with other detection frameworks, although the detection efficiency is lower, the detection precision has been improved to a certain extent.

## Conclusion and outlook

This paper proposes a local u-shape attention decoupling network (LUANets) for thermal infrared image object detection, which models the initial features captured by Res2Net-DCN, further realizes the construction of multilevel semantic information and adopts bidirectional semantic. The cross-aggregation strategy strengthens the dependency relationship between them. At the same time, it considers the rich semantics contained in the low-level physical appearance and describes the object from multiple perspectives, such as low-, middle-, and high-level, so that different types of features have a stronger relationship. The method also has the ability to discern information. In addition, in the local embedded U-shaped module, the squeeze excitation layer is used to reduce the parameters of the model, while the global attention layer better filters the key information and weakens the characterization ability of irrelevant information. Finally, evaluation and demonstration were conducted on baseline data such as FLIP, TIIs and SportFCs, and the best performance was achieved.

Although the proposed detection framework has achieved a competitive advantage, it is still found in the experimental process that there are still shortcomings, such as cross-aggregation, and it is easy to ignore the detailed information of small objects. At the same time, there is room for improvement in the parameters of the model. Therefore, in future work, we will focus on aggregating the detailed information of small-scale objects and further reducing the number of model parameters to develop a concise and efficient semantically oriented network to obtain better detection results.
